# Treating hard‐to‐heal skin and nail onychomycosis of diabetic foot with plasma therapy

**DOI:** 10.1111/dth.15127

**Published:** 2021-09-15

**Authors:** Yuta Terabe, Nobuhito Kaneko, Hiroshi Ando

**Affiliations:** ^1^ Limb Salvage Center Kasukabe Chuo General Hospital Saitama Japan

Dear Editor

Onychomycosis is a common skin and nail infection in diabetic mellitus (DM) patients. Treatments typically require topical and oral antifungals. Almost all tinea cases are cured with antifungals, but rare hard‐to‐heal infections can occur. These cases sometimes can result in an amputation. Plasma treatment is another option for tinea infections. Cold atmospheric plasma (CAP) was recently developed and has antibacterial, antifungal, and antiviral effects among others.^1^ Our service used plasma care®, made by terraplasma medical GmbH (Munich, Germany). This is the first report of the use of plasma care® in our country. Informed consent was obtained from the patient.

A 80 years old female had DM (HbA1c 8.5%) with a history of a right foot below knee amputation and a left 4th toe amputation 3 years ago. She got onychomycosis on her left foot and nail. A KOH prep was positive. Cultures grew *Trichophyton rubrum*. She started treatment with topical antifungal ointment (Efinaconazole 10% solution and Luliconazole topical ointments). The treatment for 4 weeks was unsuccessful, as was oral antifungal treatment with Fosravuconazole l‐Lysine Ethanolate (100 mg/day). The patient continued to get worse (Figure 1A). The patient therefore trialed plasma therapy. Plasma therapy treatment was performed 10 min once a day. One week later, the patient's foot had improved and her culture results were negative (Figure 1B). Her topical therapy was changed from antifungal ointment to moisture ointment with plasma therapy 2 weeks later (Figure 1C). The patient finished plasma therapy after a total of 3 weeks of treatment (Figure 1D). There were no adverse events related to plasma therapy.

**FIGURE 1:**
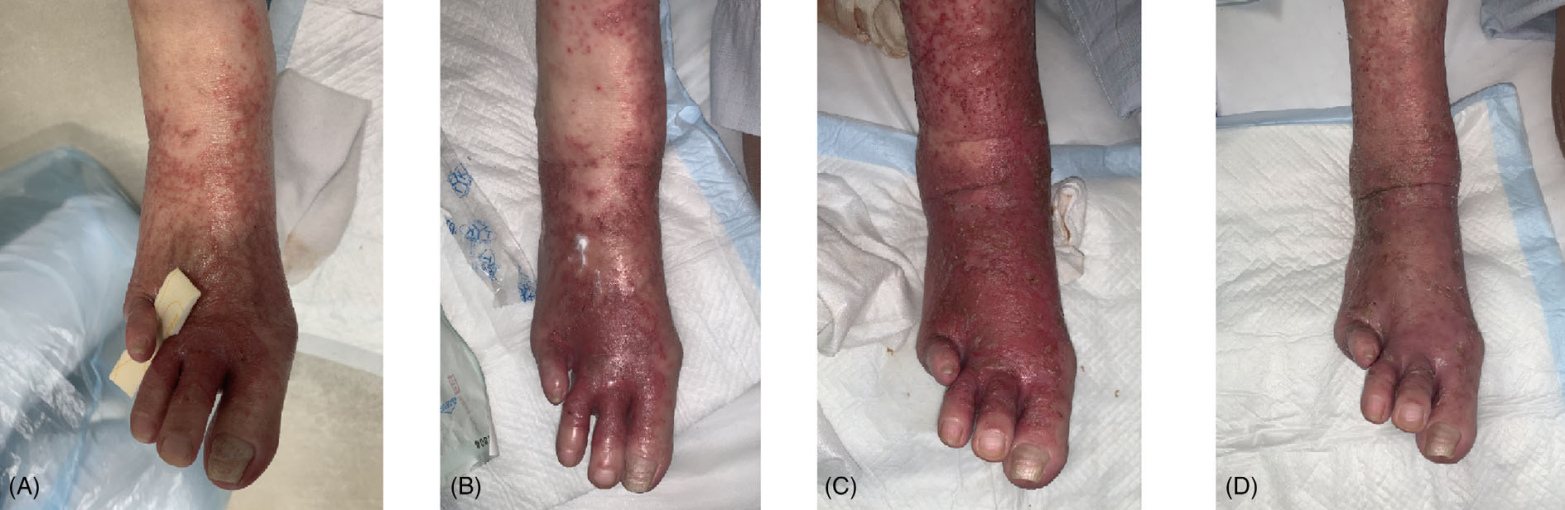
The patient's left foot. (A) Pre‐treatment. (B) After 1 week of treatment. (C) After 2 weeks of treatment. (D) After 3 weeks of treatment

The use of plasma therapy has expanded into multiple fields. In the present case, a hard‐to‐heal ulcer and skin infection were treated with plasma. Plasma has potent anti‐infection properties.^2^ There are few adverse effects associated with plasma care®, which includes mild heating and pain.

In this case, the patient had DM and her right leg had already been amputated. She feared that her left leg would require amputation, however her foot tinea was resistant to healing despite general treatment. General treatments have a low healing rate for onychomycosis. The rate of Efinaconazole solution and Fosravuconazole l‐Lysine Ethanolate capsule were 17.8% and 59.4%. Here we show that plasma treatment is a novel alternative treatment for hard‐to‐treat skin and nail onychomycosis.

Plasma has an effective in the treatment of *Trichophyton rubrum* on toe nail onychomycosis in vivo.^3^ Plasma care® is compact size and individually packaged sterile attachment to prevent cross‐contamination.^4^ This is because plasma treatment is easy to perform and has few side effects. During the first week of treatment, a para‐medical treated the patient and taught her how to use plasma care®. The patient was able to treat herself by the second week of therapy.

Plasma therapy appears to have potential efficacy against hard‐to‐heal toe nail and foot skin onychomycosis when used in conjunction with traditional treatments.

## CONFLICT OF INTEREST

The authors declare no potential conflict of interest.

## ETHICS STATEMENT

This study was approved by the research ethics committee of Kasukabe Chuo General Hospital (Permission No. 2007‐3).

## Data Availability

The data that support the findings of this study are available from the corresponding author upon reasonable request.
